# Supplementary material for: The interactome of intact mitochondria by cross-linking mass spectrometry provides evidence for coexisting respiratory supercomplexes
Source: Mol Cell Proteomics. 2017 Dec 8;17(2):216–32. doi: 10.1074/mcp.RA117.000470 (PMC5795388; doi:10.1074/mcp.RA117.000470)
Supplement: Supplemental Data [file supp_RA117.000470_133922_0_supp_23978_fzffwf.zip › spectra_annotation/mito_DR_spectra_annotation/364-1-1-1-3-1.pdf]

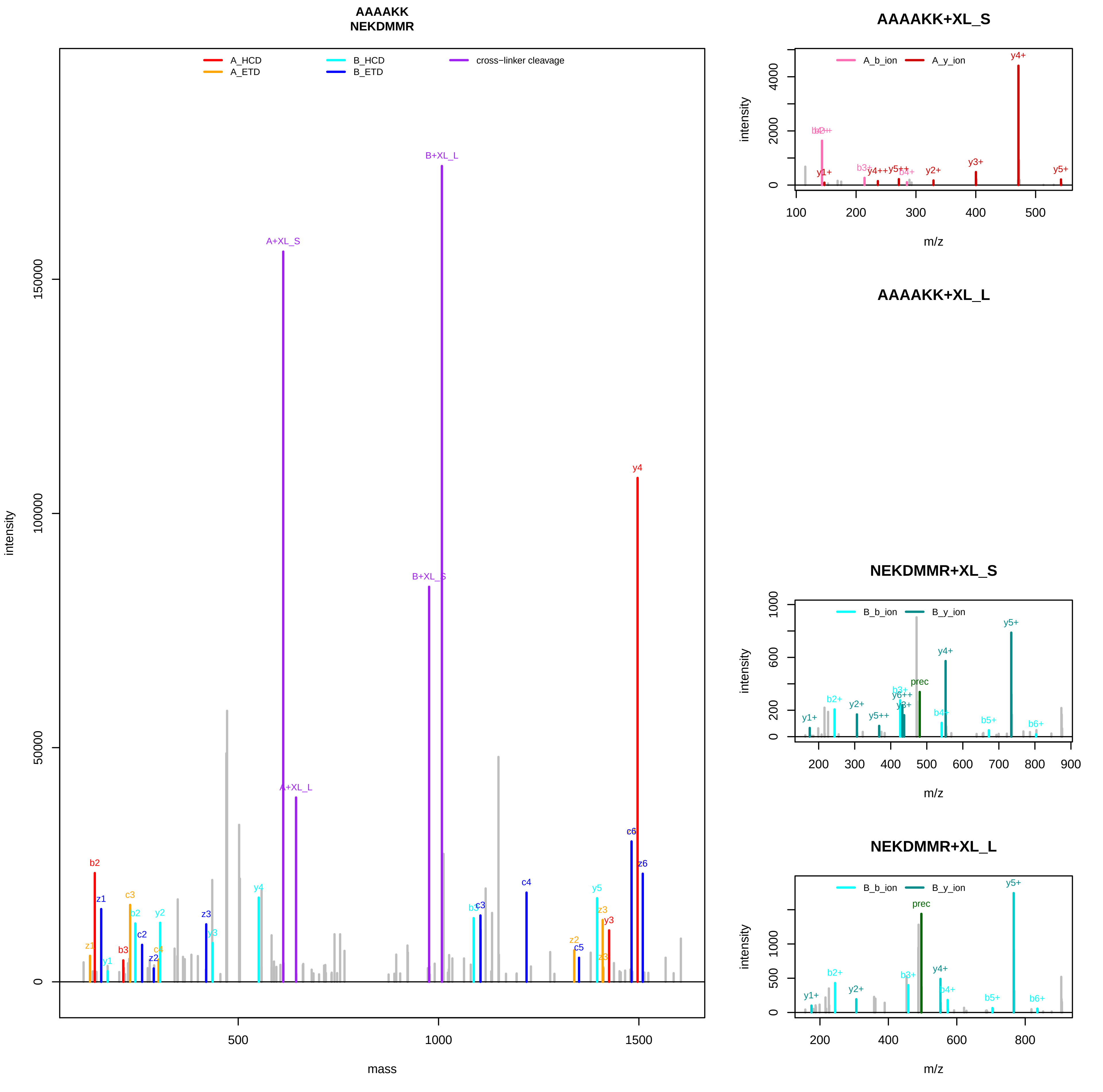
AAAAKK
NEKDMMR
A_HCD
A_ETD
B_HCD
B_ETD
cross−linker cleavage
intensity
mass
0
50000
100000
150000
500
1000
1500
B+XL_L
A+XL_S
B+XL_S
A+XL_L
y4
c6
z6
c4
y5
z3
y3
z2
c5
b3
c3
y4
z3
y3
b2
z1
c3
b2
y2
c2
z2
c4
z1
y1
b3
AAAAKK+XL_S
A_b_ion
A_y_ion
y4+
b2+
y1+
b3+
y4++
y5++
b4+
y2+
y3+
y5+
m/z
100
200
300
400
500
0
2000
4000
AAAAKK+XL_L
NEKDMMR+XL_S
B_b_ion
B_y_ion
y5+
y4+
prec
b3+
y6++
y3+
b2+
y2+
y5++
y1+
b4+
b5+
b6+
0
200
600
1000
200
300
400
500
600
700
800
900
NEKDMMR+XL_L
y5+
prec
b2+
y2+
y1+
b3+
y4+
b4+
b5+
b6+
0
500
1000
200
400
600
800
